# Treatment results in patients with ductal carcinoma in situ treated with adjuvant radiotherapy

**DOI:** 10.3906/sag-1810-53

**Published:** 2019-08-08

**Authors:** Zeliha GÜZELÖZ, Zümre ARICAN ALICIKUŞ, Nesrin AKTÜRK, Merih GÜRAY, Ali İbrahim SEVİNÇ, Pınar BALCI, İlknur BİLKAY GÖRKEN

**Affiliations:** 1 Dokuz Eylül University Medical School/Dokuz Eylül University Breast Tumor Group, İzmir Turkey; 2 Department of Radiation Oncology, Tepecik Regional Training and Research Hospital, University of Health Sciences, İzmir Turkey; 3 Department of Radiation Oncology, Faculty of Medicine, Dokuz Eylül University, İzmir Turkey; 4 Department of Pathology, Faculty of Medicine, Dokuz Eylül University, İzmir Turkey; 5 Department of General Surgery, Faculty of Medicine, Dokuz Eylül University, İzmir Turkey; 6 Department of Radiology, Faculty of Medicine, Dokuz Eylül University, İzmir Turkey

**Keywords:** Ductal carcinoma in situ, adjuvant radiotherapy, breast cancer

## Abstract

**Background/aim:**

The aim of this study was to evaluate the treatment results of patients undergoing adjuvant radiotherapy (ART) after breast surgery with the diagnosis of ductal carcinoma in situ (DCIS).

**Materials and methods:**

A total of 61 women who had undergone radiotherapy following extensive surgical excision were enrolled. All patients underwent 50 Gy ART. Survival analysis was performed using Kaplan–Meier analysis and SPSS 20.0.

**Results:**

The median age was 52 years (range: 28–86). The median follow-up period after RT was 92 months (range: 23–237). The median overall survival and distant and regional recurrence-free and disease-specific survival was 96 months (range: 26–240), while disease-free and local recurrence-free survival was 96 months (range: 22–240). While the 15-year and 20-year overall survival rates were 87% and 87%, respectively, the recurrence-free survival rates were 98% and 98%, respectively; the rates of disease-specific survival were 100% and 100%, respectively.

**Conclusion:**

The results of this study with a long follow-up period have shown that ART in DCIS is an effective treatment method to provide local disease control. However, further large studies are needed to identify the prognostic factors.

## 1. Introduction

Ductal carcinoma in situ (DCIS) constitutes 85% of noninvasive breast cancers [1]. The number of patients diagnosed with DCIS is rapidly increasing due to the more common use of mammography [2].

DCIS has risk factors similar to invasive breast cancers, such as family history, reproductive history, nulliparity, diet, and environmental factors [3]. 

DCIS is pathologically examined in 5 subtypes: comedo, cribriform, micropapillary, papillary, and solid. The comedo subtype of DCIS is thought to have a higher risk of recurrence as DCIS or invasive carcinoma than other subtypes [4,5]. Although they are noninvasive lesions, they have a risk of conversion to invasive carcinoma in about 36% of cases [6,7].

Although distant metastases are not expected in patients with DCIS, the main problem in follow-up is local recurrences. Poor prognostic factors increase the risk of recurrence [8].

In the last 30 years, with the addition of adjuvant radiotherapy (ART) to breast-conserving surgery (BCS) in early-stage invasive tumors, the organ has been successfully preserved [9–11]. This success in invasive tumors has resulted in the interpretation of mastectomy as an excessively radical surgery in DCIS, a noninvasive form of breast cancer.

In a Cochrane metaanalysis comparing four large-scale, randomized, controlled trials, it was reported that recurrence in the ipsilateral breast (HR = 0.49) and DCIS recurrences (HR = 0.64) had occurred in 3925 patients who had undergone BCS + ART and that all subgroups had benefited from RT [12]. Although local recurrences were reported at a higher rate in cases of BCS compared to mastectomy, there was no difference in survival between the two treatment modalities [9–11].

According to the results of four large-scale, randomized, controlled trials with patients with DCIS, RT was reported to halve the local recurrence rates. Radiotherapy reduces the local recurrence rate from 16%–22% to 7%–10%. Therefore, local treatments are preferred in DCIS management [13–16].

Satisfactory outcomes with current treatment modalities of BCS together with ART applications have shifted the management of DCIS towards broad excision + ART rather than mastectomy. Local recurrences vary depending on multiple factors such as tumor size, grade, surgical margin (SM), and presence of adjuvant hormonal therapy administration. The 5-year local recurrence rates have been reported as 16% [17]. According to randomized controlled studies, this local recurrence rate can be reduced to 3%–8% with ART [13–16].

The Van Nuys Prognostic Index (VNPI) was developed by retrospective evaluation of patients undergoing ART following BCS in a single center. Patients are classified into low-, moderate-, and high-risk groups using parameters such as age, histological grade, SM characteristics, and tumor size, and a treatment choice is recommended according to the risk category [18].

 In this study, we aimed to evaluate the long-term results of treatment in patients with DCIS who had undergone adjuvant radiotherapy following VNPI surgery within the scope of the Dokuz Eylül University Breast Tumors Group (DEMTG) breast cancer treatment protocol. 

## 2. Materials and methods

### 2.1. Patients

In this study, the medical archive of the Department of Radiation Oncology was reviewed retrospectively. A total of 61 patients who had undergone adjuvant RT between January 1992 and January 2016 with the diagnosis of DCIS were included in the study.

### 2.2. Treatment

In our clinic, after having discussed the cases in the Breast Tumor Group, BCS is applied to patients who have a cosmetically suitable breast/tumor ratio, who are not pregnant in the first trimester, who do not have widespread microcalcifications on mammography, who do not have a tumor in multiple quadrants, who do not have a history of previous RT to the chest wall, and who do not have active collagen tissue disorder. Patients with early-stage breast cancer and DCIS who meet these criteria undergo wide excision. Reexcision is performed in the presence of a positive or near surgical margin (<2 mm). Sentinel lymph node sampling is performed for patients with high-grade tumors and comedo-necrosis according to the pathology results. Until 9 years ago, the radiotherapy indication was decided according to the patients’ VNPI score. However, ART has been given to every patient who underwent extensive excision and who was diagnosed with DCIS since then. After ART, tamoxifen at 20 mg/day is given for 5 years according to the hormone receptor status.

The patients were irradiated in the supine position using a mammary board. Treatment planning was performed with 3-section tomography images by a conventional simulator between 1992 and 2003, while after 2003, a 3-dimensional conformal RT technique was

used. Two tangential areas using high-energy photons (CO-60 or 6 MVX) for the entire breast were used in ART. 

### 2.3. Follow-up 

Patients were evaluated once a week during RT at the outpatient clinic. Thereafter, they were called for a follow-up visit once every 3 months during the first 2 years after RT, every 6 months between 2 and 5 years, and annually after 5 years. At the follow-up visits, patients underwent a thorough physical examination and were followed with annual mammography. The early and late side effects of RT were evaluated according to the RTOG/EORTC criteria.

At follow-up, cosmetic results were scored subjectively on a scale of 1–5 points. One point indicated a very poor cosmetic result, while 5 points indicated a breast with similar cosmesis to a healthy breast despite the treatments applied.

### 2.4. Statistical analysis 

Survival analyses were performed using Kaplan–Meier survival curves and the prognostic value of the variables was determined by log-rank and Cox regression tests using SPSS. The parameters related to the local control were calculated from the date of completion of RT and the overall survival time was calculated from the date of diagnosis.

## 3. Results 

The median age of the patients was 52 years (range: 28–86). The most common tumor localization was in the right breast (62%) and upper outer quadrant (48%). Forty-eight (79%) patients were diagnosed by mammography. Eight of the patients presented with a palpable mass in the breast, one of them had bloody discharge, and one had colorless-serous discharge.

Wide excision was performed on all patients. Five (8%) patients underwent axillary intervention in the form of sentinel lymph node biopsy. There was no axillary metastasis in any patient.

As shown in the Table, the tumor size was ≤15 mm in 66% of patients, 16–40 mm in 33%, and ≥40 mm in 2%. Fifty-two (85%) patients had negative surgical margins. In terms of nuclear grading, 8 (13%) patients had grade 1, 31 (51%) had grade 2, and 17 (28%) had grade 3 tumors, while the grades of 5 (8%) patients were not identified. Comedo necrosis was present in 56% of the patients. According to the VNPI scores, 30% of the patients were evaluated as 4–6 points, 62% as 7–9 points, and 8% as 10–12 points.

**Table:** Demographic and pathologic data.

Characteristics	Patient number (%)
Age ≥6040–59≤40	11 (24%)33 (72%)2 (4%)
Nuclear gradeGrade 1Grade 2Grade 3Unknown	8 (13%)31 (52%)17 (27%)5 (8%)
Tumor size≤15 mm15–40 mm≥40 mm	40 (66%)20 (33%)1 (2%)

A total of 50 Gy per 2 Gy fraction was applied daily to all patients with conventional fractionation, while a total dose of 60 Gy ART was applied to 31 (51%) patients, including a boost to the tumor bed. 

Estrogen receptor was positive in 77% of patients and progesterone receptor was positive in 71% of the patients; all receptor-positive patients used tamoxifen at 20 mg/day after RT.

The median follow-up period was 97 months (range: 26–240) after RT. Local recurrence was detected in only one patient (3%) at the 19th month, and no regional or distant metastasis was observed in any of the patients. The patient with local recurrence had undergone simple mastectomy and histopathological examination revealed invasive ductal carcinoma. This patient had received adjuvant chemotherapy and is still being followed without any disease for 129 months after the local recurrence.

The median local recurrence-free survival was 96 months (range: 22–240), while the disease-specific survival and overall survival (OS) was 96 months (range: 26–240). Fifteen and 20-year local recurrence-free survival rates were 98% and 98%, disease-specific survival rates were 100% and 100%, and OS rates were 87% and 87%, respectively.

Ten (16%) patients developed a non-breast cancer synchronous tumor. Due to the small number of events, statistical analysis could only be carried out for OS, which showed that older age and presence of a second primary tumor had a negative effect on the overall survival.

None of the patients had grade 3–4 side effects according to the RTOG/EORTC Late Radiation Morbidity Scoring Schema [19]. All patients had grade 1 skin and subcutaneous side effects. Cosmetic evaluation was performed for 46 patients according to the Modified Harvard–Harris Scale of Breast Cosmesis [20]. Twelve (20%) patients scored 2/4 points, 23 (38%) patients had 3/4 points, and 11 (18%) had 4/4 points.

## 4. Discussion 

DCIS consists of proliferated malignant epithelial cells of the breast ducts that have not crossed the basal membrane. It is considered a transition from normal breast tissue to carcinoma. It takes place in a wide spectrum of transformation risk, from low-grade lesions to high-grade invasive tumors. Although it was a rarely seen breast pathology in the 1980s, it currently accounts for 25% of breast cancers in the United States thanks to widespread screening programs [21].

Mastectomy had been defined as the standard treatment option for the first 40 years after it was first defined in the literature. Locoregional recurrences are rare after mastectomy [22]. There is no randomized trial comparing mastectomy with BCS. In nonrandomized studies, mastectomy was found to be superior to BCS in terms of local control, but there was no difference in survival [23]. BCS and ART are now recommended as standard therapy [17,24].

In our study, 15- and 20-year distant metastasis-free and disease-specific survival rates were found to be 100% in patients with DCIS undergoing BCS and receiving ART. However, the main problem in patients with DCIS is ensuring local disease control [25]. The success of surgery in local control has increased with the addition of ART. In the ECOG 5194 study, Motvani et al. compared ipsilateral breast tumor development rates in low-risk DCIS patients undergoing ART after BCS with those who did not. Even in the low-risk groups, the addition of adjuvant RT significantly decreased ipsilateral breast tumor formation [26]. In their 818-patient series, Fisher et al. compared lumpectomy-only with lumpectomy followed by 50 Gy ART and reported that the local failure rate in noninvasive disease decreased from 15% to 8% in the RT-receiving arm [13]. In the study of Holmberg et al., local failure in the noninvasive group decreased from 13% to 4% with ART administration [16]. In the UKCCR study, RT administration was found to reduce the rate of local failure in the invasive and noninvasive ipsilateral breast from 14% to 6% [15]. As a result, previous studies that compared BCS alone with BCS + ART found that the rate of local recurrence decreased by half with the addition of radiotherapy to the treatment, regardless of the effects of age, SM status, presence of comedo necrosis, nuclear grade, and tamoxifen use, and this effect in particular was more prominent between the ages of 40 and 59 years.

With today’s technology, treatments are individualized. The Oncotype Dx DCIS score, generated by 12-gene test scoring for DCIS, provides 10-year prognostic and predictive information for patients, which categorizes the patients into low-, medium-, and high-risk groups. This test, which has not yet been introduced into daily clinical practice, is expected to cause changes in the indications for RT in patients with DCIS over time [27].

 As of yet, there is no prospective study on boost application to the tumor bed after ART in DCIS patients undergoing BCS. BONBIS and TROG 07.01 are the only ongoing prospective randomized control clinical trials looking for the role of boost application in DCIS.ClinicalTrials.gov., Breast-Conserving Surgery and Whole-Breast Radiation Therapy With or Without Additional Radiation Therapy to the Tumor in Treating Women With Ductal Carcinoma in Situ, NCT00907868. https://clinicaltrials.gov/ct2/NCT00907868.  ClinicalTrials.gov., Radiation Doses and Fractionation Schedules in Non-Low Risk Ductal Carcinoma In Situ (DCIS) of the Breast (DCIS), NCT00470236. https://clinicaltrials.gov/ct2/NCT00470236. However, there are some studies with patients having early-stage invasive breast carcinoma. In the EORTC Boost study, the patients who received 50 Gy ART were compared with those receiving 50 Gy + 16 Gy boost doses. In 10 years of follow-up, the local failure rate was found to decrease from 10.2% to 6.2% in the boost-receiving group and it was emphasized that this effect was more apparent in women aged 40 years or younger [28]. In the Lyon study, the local failure rate decreased from 4.5% to 3.6% upon 3-year follow-up with the addition of a 10 Gy boost dose to the tumor bed in invasive breast carcinoma patients [29]. There are some retrospective data showing the role of an additional dose as a boost application for patients treated with adjuvant RT for DCIS. These studies show that patients with positive SM, comedo necrosis, and unknown receptor status had received a boost dose and that led to a reduction in ipsilateral breast recurrence rates [30,31]. In our study, all patients were given adjuvant RT after BCS and 31 patients were given an additional boost to the tumor bed. The local control rate was found to be 98% in accordance with the literature. Only one patient had local recurrence. This patient had a tumor size of 18 mm, was in the moderate-risk group, and had comedo necrosis.

Sentinel lymph node sampling is not routinely recommended in patients with DCIS. Sentinel lymph node sampling is recommended in high-risk patients with suspicion of an invasive tumor or large tumor size that requires mastectomy [32]. In our study, sentinel lymph node sampling was performed for 5 patients.

In previous randomized trials, tamoxifen therapy combined with RT was found to reduce the risk of local recurrence. In the study of Fisher et al. with a total of 1009 lymph node-negative invasive breast carcinoma patients, the recurrence rate was 16.5% in the tamoxifen-only arm, while the recurrence rate in the RT + tamoxifen arm had decreased to 2.8% [33]. In the NSABP B-24 study, the addition of tamoxifen to radiotherapy following BCS decreased the rate of breast cancer events from 13.4% to 8.2% [34]. In our study, 49 patients who were receptor-positive received adjuvant tamoxifen treatment for 5 years.

The first hypofractionation study of patients with invasive breast cancer was the START A trial, which was followed by START B and the Canada study. All three studies showed similar results for both local control and cosmetic outcome rates with the hypofractionated RT raising the question of whether this could be implemented in ART for DCIS [35–37]. In our study, RT was administered to patients with a conventional fractionation scheme. In the literature, the results of patient groups treated with hypofractionated RT in DCIS have also been reported.

In the study of Lalani et al., hypofractionated RT (42.4 Gy in 16 fractions) was compared with conventional fractionation (50 Gy in 25 fractions); 60% of the patients were treated conventionally and the remainder with a hypofractionation scheme. The 10-year local recurrence-free survival rates were 86% and 89% for the conventional and hypofractionation schemes, respectively (P = 0.03). Multivariate analysis showed no increase in local failure rates with the hypofractionation scheme in patients with DCIS diagnosis [38]. Wai et al. used a hypofractionation scheme (44 Gy in 16 fractions) in 77% of the patients in their study, which had 957 DCIS patients. There was no difference in local control rates between the hypofractionation and conventional fractionation schemes within the 9.5-year follow-up period [39]. In the hypofractionated RT study of Williamson et al. on DCIS patients, they compared the conventional and hypofractional RT regimens. In this study, the 4-year recurrence risks were reported as 6% and 4%, respectively [40]. In all three studies on invasive breast carcinoma, there was no difference in terms of local control and cosmetic results. These hypofractionation schemes became standard management in invasive carcinomas after these studies. However, the standard approach is still in the form of conventional fractionation in patients with DCIS considering that the follow-up period is shorter in hypofractional applications in DCIS and they were conducted in single centers with a relatively lower number of patients to evaluate the long-term local control and cosmetic results.

In conclusion, the administration of ART in patients undergoing breast surgery with the diagnosis of DCIS is an effective treatment method for local disease control. In our study, the number of patients was insufficient to detect the prognostic factors affecting recurrence-free survival. Further large-scale studies with longer follow-up periods are needed to clarify this issue.
